# Clinical Risk Factors for Aortic Root Dilation in Patients with 22q11.2 Deletion Syndrome: A Longitudinal Single-Center Study

**DOI:** 10.3390/genes13122334

**Published:** 2022-12-10

**Authors:** Carolina Putotto, Federica Pulvirenti, Flaminia Pugnaloni, Ina Isufi, Marta Unolt, Silvia Anaclerio, Viviana Caputo, Laura Bernardini, Elisa Messina, Corrado Moretti, Luigi Tarani, Bruno Marino, Paolo Versacci

**Affiliations:** 1 Department of Maternal Infantile and Urological Sciences, Sapienza University of Rome, 00161 Rome, Italy; 2 Regional Reference Centre for Primary Immune Deficiencies, Azienda Ospedaliera Universitaria Policlinico Umberto I, 00185 Rome, Italy; 3 Neonatal Intensive Care Unit, Medical and Surgical Department of Fetus, Newborn and Infant—Bambino Gesù Children’s Hospital IRCCS, 00165 Rome, Italy; 4 Department of Pediatric Cardiology and Cardiac Surgery, Bambino Gesù Children’s Hospital IRCCS, 00165 Rome, Italy; 5 Department of Experimental Medicine, Sapienza University of Rome, 00161 Rome, Italy; 6 Cytogenetics Unit, Casa Sollievo della Sofferenza Foundation, San Giovanni Rotondo, 71013 Foggia, Italy

**Keywords:** 22q11.2 deletion syndrome, aortic root dilation, clinical risk factors, skeletal and connective tissue disorders, longitudinal course, periodic cardiac evaluation

## Abstract

Background: Aortic root dilation (ARD) has been described in 22q11.2DS, even without congenital heart disease (CHD). However, the clinical implications and longitudinal course are unclear. In this study, we evaluated aortic root (AR) dimensions in 22q112.DS adolescents/adults without major intracardiac CHDs, analyzed the progression over time and investigated correlations with extracardiac comorbidities. Methods: AR dimensions were evaluated in 74 patients, measuring the sinus of Valsalva (VS) and proximal ascending aorta (AA), using Z-score to define mild, moderate and severe degrees. Changes in AR dimensions during longitudinal echocardiographic follow-up were investigated. Phenotypic characteristics have been collected. Results: Twenty-four patients (32.4%) showed ARD in terms of VS Z-score (2.43; IQR 2.08–3.01), eight (33.3%) of a moderate/severe degree. Thirteen (54.2%) had concomitant AAD (Z-score 2.34; IQR 1.60–2.85). The risk of ARD was significantly directly related to skeletal/connective tissue disorders (OR 12.82, 95% CI 1.43–115.31; *p* = 0.023) and inversely related to BMI (OR 0.86, 95% CI 0.77–0.97; *p* = 0.011). A significant increase in AR diameter’s absolute value (*p* = 0.001) over time has been detected. Conclusion: Isolated ARD is common in 22q11.2DS. Although some clinical risk factors have been identified, pathogenetic mechanisms and risk of complications are undefined. Regular cardiac evaluations should be part of the 22q11.2DS follow-up, and also in non-CHDs patients, to improve long-term outcome.

## 1. Introduction

The 22q11.2 deletion syndrome (22q11.2DS) (OMIM#188400/192430) is considered the most frequent chromosomal microdeletion disorder in humans, showing an incidence of about 1/4000 live births and 1/1000 fetuses and it is characterized by a wide and heterogeneous range of clinical features [1]. Almost 75–80% of patients with 22q11.2DS have a congenital heart defect (CHD) although the exact prevalence is still unknown [2]. Many studies in the last 30 years have elucidated an interesting genotype–cardiac phenotype correlation, demonstrating that 22q11.2DS patients mainly display CHD due to outflow tract disruption such as conotruncal heart defects, including tetralogy of Fallot (ToF), pulmonary atresia (PA), truncus arteriosus (TA), ventricular septal defect (VSD), interrupted aortic arch type B (IAA-B), aortic arch anomalies (such as right sided aortic arch—RAA, double aortic arch—DAA), abnormal branching of the epiaortic vessels and crossed pulmonary arteries [3,4,5,6]. Less frequently, 22q11.2DS patients may present with other CHDs and a few studies have reported that aortic root dilation (ARD), with or without CHD, can be a cardiac feature among the pediatric and young adult 22q11.2DS population [7,8,9].

Progressive ARD has been extensively described in patients with repaired and unrepaired CHD and in the setting of specific genetic syndromes (i.e., Marfan syndrome, Ehlers–Danlos syndrome, Loeys–Dietz syndrome, Turner syndrome) [10,11,12] whereas there is still a paucity of data assessing ARD in the context of 22q11.2DS. Interestingly, some of these known syndromic connective tissue disorders, caused by molecular defects in genes related to extracellular matrix synthesis and remodeling, result in multisystem involvement, especially of the cardiovascular and skeletal systems, which are also frequently involved in the clinical phenotype of 22q112DS [13]. Currently, the pathogenesis, the natural history, the functional significance and the long-term outcome for isolated ARD in 22q11.2DS remain undefined.

Due to the growing number of patients with 22q11.2DS surviving into adulthood [14] and to the great improvements in surgical outcome and cardiac imaging, a better understanding of the prevalence and the underlying pathophysiology of ARD is warranted.

The aim of our study is therefore to describe the prevalence of ARD in our single-center cohort of adolescents and adults with 22q11.2DS and to provide insights about clinical risk factors associated with ARD, in particular, cardiovascular risk factors (such as dyslipidemia, hypertension, overweight/obesity, diabetes mellitus type 2—DMT2 and smoking), aortic arch and/or epiaortic vessel anomalies and skeletal and connective tissue disorders. Moreover, we evaluated the longitudinal course of ARD over the entire period of patients’ follow-up.

Our study may offer clinically valuable information to incorporate in multidisciplinary management with potential long-term implications for prevention and prognosis in these patients.

## 2. Materials and Methods

### 2.1. Patients

Patients ≥16 years of age with a confirmed genetic diagnosis of 22q11.2DS were evaluated for the enrollment at the Department of Maternal Infantile and Urological Sciences of Sapienza University of Rome, which is the largest Italian referral care center for the diagnosis and treatment of adolescents and adults with 22q11.2DS. From a total cohort of 125 patients, 47 individuals with major intracardiac CHDs (ToF, PA, TA, VSD and IAA) were excluded. Among 78 patients with 22q11.2DS without major intracardiac CHDs, 74 individuals, who had undergone ≥1 transthoracic echocardiogram as a part of their regular multispecialty follow-up from 2015 to 2021, were included in the analysis. Aortic root (AR) size was abstracted from 104 echocardiograms performed, with 14 patients having more than one echocardiogram. Patients with minor cardiac structural abnormalities, such as aortic arch anomalies (DAA and RAA) with/without epiaortic vessel anomalies, Kommerell diverticulum (i.e., an aneurysmatic origin of an aberrant left or right subclavian artery), crossed pulmonary arteries and vascular ring were enrolled in the study. We also included patients with bicuspid aortic valve (BAV).

The study complies with the Declaration of Helsinki and was approved by our local Institutional Review Boards for Human Studies (Protocol Ref. 5803).

Demographic and anthropometric characteristics (sex, body mass index—BMI), cardiovascular risk factors (hypertension, dyslipidemia, overweight/obesity, DMT2, smoking) and orthopedic and connective tissue disorders (defined as the presence of at least one congenital abnormality of the upper and lower limbs, congenital vertebral anomaly, scoliosis, ligamentous laxity) were collected for the whole cohort of patients.

Genetic aspects of our cohort of 22q11.2DS patients were investigated, including, when available, the position and the extension of the 22q11.2 deleted region and the presence of additional copy number variants (CNVs), besides the known 22q11.2 microdeletion [15]. The genetic data were aligned following the human reference genome (GRCh37/hg19) and the identification of the specific genomic position of the microdeletion was analyzed referring to the genomic clusters of chromosome 22 (from LCR22-A to LCR22-H) [16].

Proximal aortic diameter assessment. Proximal aortic diameters were measured in 2-dimensional (2D) parasternal long axis view in diastole at 2 levels: sinus of Valsalva (VS) and proximal segment of the ascending aorta (AA). The echocardiographic parameters were standardized for the BSA as calculated by means of the Haycock formula. According to guidelines [17,18], a Z-score analysis was used to define mild (Z-score 2–3), moderate (Z-score 3.01–4) and severe (Z-score > 4) ARD. We analyzed all the echocardiographic exams that patients underwent periodically in our institution throughout their entire follow-up every 1–2 years to investigate the progression of AR dimensions over time. All the echocardiographic exams were performed by the same physician. To exclude any potential bias, a second physician who was unaware of the patients’ genetic status analyzed the echocardiographic records off-line.

### 2.2. Statistical Analysis

The primary analysis aimed to investigate clinical, demographic and genetic characteristics in two groups defined as ARD vs. non-ARD 22q11.2DS patients. Continuous variables were described using median and interquartile ranges, and categorical variables using frequencies and percentages. Group differences were analyzed by the Wilcoxon rank sum test to compare continuous variables for non-normally distributed data and by Student’s *t*-test for normally distributed data; the χ^2^ test was used for categorical variables. A *p* value of <0.05 was used to consider differences statistically significant. Secondary analysis was performed to ascertain risk factors associated with ARD diagnosis using the Kruskal–Wallis test or analysis of variance (ANOVA). We performed a multiple logistic regression model using only statistically significant variables identified with univariate analyses, except the variable AAD that has been supposed to be influenced by the same independent variables as ARD. Odds ratios and 95% confidence intervals (CIs) were calculated. For repeated echocardiogram exams, a two-way ANOVA test was used to analyze changes in AR diameter expressed both as Z-score and as absolute value (expressed in millimeters—mm) and changes in BMI over time. Since few participants had more than two exams, comparisons between the first and the second echocardiograms were also performed by a Wilcoxon rank sum test (for non-normally distributed data) or Student’s *t*-test. Statistical analyses were performed with SPSS 18.0 software for Windows (SPSS, Chicago, IL, USA) and GraphPad Prism version 8.0.0 for Windows, GraphPad Software, San Diego, CA USA.

## 3. Results

### 3.1. Patients and Demographics

Among 74 22q11.2DS participants (age 27.5 years (IQR 23–35), males 44 (59.5%)), twenty patients (27%) had aortic arch/epiaortic vessel anomalies, nineteen (25.7%) showed crossed pulmonary arteries and four patients (5.4%) had BAV. The median value (IQR) of BMI was 23.7 Kg/m^2^ (20.5–29.1). Other baseline characteristics are reported in Table 1.

Twenty-four patients (32.4%) were found to have ARD seen at the screening echocardiogram. In the ARD group, the median (IQR) of the VS Z-score at the first altered echocardiogram was 2.43 (2.08–3.01) and the highest Z-score recorded was 4.21. Eight individuals (33.3% among the total patients with ARD) displayed a moderate/severe ARD, including two with a Z-score > 4.

Nineteen patients (25.7%) showed a dilation of the ascending aorta (AAD) (Z-score 2.7; IQR 2.4–3.2). Six (31.6% of the total patients with AAD) had a moderate/severe degree, including two patients with a Z-score > 4 and one patient with a Z-score > 5.

Thirteen out of twenty-four patients (54.2%) had ARD associated with AAD, with ARD and AAD being significantly related (*p* < 0.0001). Among those having ARD, the median (IQR) of the AA Z-score was 2.34 (1.60–2.80) (Table 2). None of the patients with ARD had cardiovascular complications during the study period. Only three patients with ARD presented a mild aortic valve insufficiency, not hemodynamically significant.

### 3.2. Comparison between ARD and Non-ARD 22q11.2DS Patients

The between-group differences among AAD and non-AAD 22q11.2DS patients are shown in Table 1. No differences in term of sex, age, hypertension, dyslipidemia, smoking and DMT2 were found to be statistically significant. Moreover, groups did not differ in having crossed pulmonary arteries or BAV. On the other hand, aortic arch/epiaortic vessel anomalies and skeletal and connective tissue disorders were significantly different between the two groups (*p* = 0.012 and *p* = 0.012). Compared with patients without ARD, patients with ARD had lower BMI values: 21.4 Kg/m² (20.1–25.4) vs. 22.2 Kg/m² (21.9–32.3), *p* = 0.005. 

### 3.3. Genetic Aspects

Table 3 shows the genetic data of our total cohort of 22q11.2DS patients. Among 74 individuals, 47 (63.5%) were diagnosed by SNP-/CGH-array, 1 (1.4%) by MLPA and 26 (35.1%) by FISH. Of these subjects, 43 patients (89.6%) presented a proximal deletion, 3 (6.3%) a distal deletion and only 2 (4.2%) exhibited a central deletion. Regarding the extension of the deletion region, 35 patients (72.9%) had a typical size of 2.5–3 Mb, while 13 patients (27.1%) showed an atypical nested extension <2.5 Mb. Among the group of individuals detected by SNP-/CGH-array, additional CNVs, besides the 22q11.2 microdeletion, were found in 11 patients (23.4%), of whom only 3 showed ARD (6%). When comparing ARD vs. non-ARD 22q11.2DS patients, there was no difference in the position or the size of the 22q11.2 deleted region and in the incidence of additional CNVs identified by SNP-/CGH-array (Table 3). Clinical, genetic and echocardiographic data of 22q11.2DS patients with ARD are summarized in Table S1.

### 3.4. Multiple Logistic Regression Analysis

A multiple logistic regression model using a stepwise selection procedure was calculated. Odds ratios and 95% confidence intervals (CIs) of multivariate models are reported in Table 3. The risk of ARD was significantly directly related to skeletal and connective tissue disorders (OR 12.82, 95% CI 1.43–115.31; *p* = 0.023) and inversely related to BMI (OR 0.86, 95% CI 0.77–0.97; *p* = 0.011). Otherwise, no relationship between the risk of ARD and the presence of aortic arch/epiaortic vessel anomalies has been detected (OR 3.02, 95% CI 0.89–10.27; *p* = 0.076) (Table 4).

### 3.5. Changes in Aortic Root Size over Time

**Figure 1:**
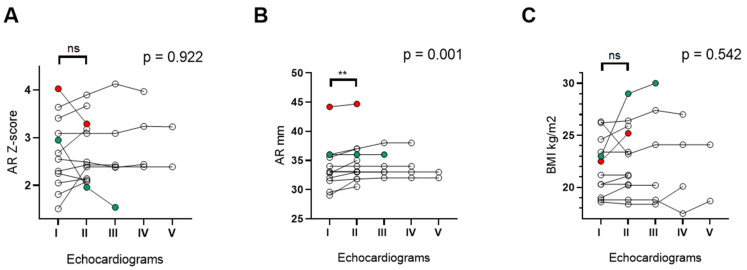
Longitudinal course of Z-score (panel **A**), absolute values of AR diameter (panel **B**) and changes in BMI (panel **C**) over the time of follow-up. The two-way ANOVA test was used to evaluate changes in AR and BMI over time (reported as *p* values). Wilcoxon matched pair signed-rank test was used to compare the first and second echocardiograms. Significant *p* values are shown as ** (*p* < 0.01). AR: aortic root, ns: not significant, BMI: body mass index.

Among 24 patients with ARD, 14 underwent at least two echocardiographic exams during the follow-up (median age 21.5 years (IQR: 19.2–25.5), males 7 (50%)). The median interval time between the first and the last echocardiogram was 2 years (IQR: 2–4). Changes in AR dimension were evaluated both by Z-score and by diameter’s absolute value (in mm). When considering Z-score, seven patients (50%) showed an increased value of AR dimension from the first and the second echocardiogram, five remained stable and two patients had, respectively, a reduction of 18% (red dots) and 33% (green dots) of their initial value (Figure 1A).

Differently, all patients showed an increased value of AR dimension when expressed as diameter absolute value, with a significant difference recorded one/two years after the first examination (I echoc. 31.6 mm (IQR: 31.6–35.9) vs. II echoc. 33.5 mm (IQR: 32.1–36.7); *p* = 0.0078)) (Figure 1B). Changes in BMI are reported in Figure 1C. An increased BMI was recorded in those patients with decreased Z-scores at the second echocardiogram (Figure 1C, red and green dots).

Changes in AR Z-score, AR diameter and BMI were also analyzed by a two-way ANOVA test, revealing a significant increase only in the absolute value of the AR diameter (*p* = 0.001) over the time of follow-up.

## 4. Discussion

ARD is typically associated with other specific known genetic syndromes characterized by a connective tissue disruption, such as Marfan, Loeys–Dietz or Ehlers–Danlos [10]. In addition, some CHDs, such as ToF, are known to have an increased risk of developing a progressive ARD over time, especially when associated with pulmonary valve abnormalities and aortic arch anomalies (such as RAA) [8,12]. However, there are few data on isolated ARD in patients with 22q11.2DS and the natural history and the long-term outcome of this finding still remain variable and unclear. Moreover, to date, the pathogenesis and the possible correlations between ARD and extracardiac phenotype of patients with 22q11.2DS have not yet been investigated.

According to literature data, our study showed that adults with 22q11.2DS without major intracardiac CHDs can present ARD of different degrees, highlighting a remarkable prevalence compared to the previous reports on pediatric and young adults cohorts of patients (32.4% vs. 16.3% vs. 10%) [7,9].

This study is the first that analyzed in detail the possible relationship between isolated ARD and some clinical characteristics in a cohort of adolescents and adults with 22q11.2DS. Cardiovascular risk factors (such as hypertension, overweight/obesity, dyslipidemia, DMT2, smoking) and aortic arch/epiaortic vessels anomalies, typical features of the 22q11.2DS-related phenotype, were evaluated.

Furthermore, considering the well-known close cardiovascular and skeletal involvement common to syndromic connective tissue disorders [13], we investigated whether there was a correlation between ARD and the presence of skeletal and connective tissue anomalies, another phenotypic finding also frequently observed in the 22q11.2DS population.

In our cohort, neither sex nor age was found to be significantly related to the presence of ARD. Moreover, none of the considered cardiovascular risk factors had a significant relationship with ARD. While univariate analysis showed a significant correlation between ARD and aortic arch/epiaortic arch abnormalities, multiple logistic regression did not confirm this finding as a risk factor for ARD, according to data previously reported in the literature [9].

Interestingly, the risk of ARD was directly related to the presence of skeletal and connective tissue abnormalities. Although our study group is limited and our results should be validated on larger cohorts, the significant relationship between the presence of ARD and skeletal and connective tissue anomalies might suggest that pathogenetic mechanisms similar to those of hereditary connective tissue diseases are also involved in this genetic syndrome, due to genes whose alteration leads to multisystem involvement, especially affecting cardiovascular and musculoskeletal systems.

To date, it is known that the extracellular matrix (ECM) has a major functional role in ensuring the integrity and function of various connective tissues (such as bone, cartilage and blood vessels) and is implicated in the etiopathogenesis of hereditary connective tissue disorders (such as Ehlers–Danlos syndrome) [13,19]. We cannot exclude that similar pathomechanisms may also occur in 22q11.2DS. A recent study on mouse models showed that *Tbx1*, a gene mainly involved in the phenotypic expression of 22q11.2DS, is required for ECM interactions and integrin adhesion, which are critical in the early stages of cardiac outflow tract morphogenesis [20].

It is interesting to note that one of our patients with ARD also showed RAA with aberrant left subclavian artery, scoliosis, vertebral anomalies and two splenic artery aneurysms, found on a routine abdomen ultrasound. In addition to ARD, another thing that could suggest the involvement of pathogenetic mechanisms implicated in the global formation of vessels in 22q11.2DS patients is the possible presence of vascular anomalies of the neck vessels, frequently observed in this population (e.g., medial deviation of internal carotid arteries, anomalous bifurcation/origin or tortuosity of the carotids) [21]. In fact, it has been shown that vascular endothelial growth factor (*VEGF*), although not included in the critical region 22q11.2, interacts with *Tbx1* as a modifier of congenital cardiovascular defects in mouse models, suggesting a downstream genomic interplay in vessels’ embryogenesis in 22q11.2DS [22].

Although a recent analysis on a cohort of 22q11.2DS patients under 25 years of age reported that AR Z-scores decreased across all levels over time and may become a minor problem during growth [8], our study showed that 50% of patients had an increase in the AR dimension in terms of Z-score and five subjects remained stable between the first two echocardiograms. The only two patients with a reduction in AR Z-scores had a significant weight increase with a consequent rise in BMI and BSA, explaining the reduced AR Z-score.

On the other hand, patients showed a statistically significant increase in the absolute values of AR diameter over time. These data suggest that, although it is useful to use a Z-score standardization in the daily routine to determine the possible presence of ARD, it is essential to take into account that this parameter is strictly dependent on the BSA, to avoid any errors of evaluation. For this reason, a high weight gain can cause a reduction in the Z-score value at the next check-up, while the patient maintains the absolute value of the AR diameter or it is even increased.

Except for some specific categories of patients who have associated conditions with an increased risk of aortic rupture/dissection that require lower threshold values (e.g., patients with BAV, Marfan syndrome, Loeys–Dietz syndrome, Ehlers–Danlos syndrome, Turner syndrome), the general guidelines recommend surgical intervention when the AR and the AA reach 55 mm and when the descending aorta reaches 60 mm [23,24].

None of our patients showed AR dissection or other cardiovascular symptoms related to ARD during the period of follow-up. At present, the severity and frequency of ARD complications in the 22q11.2DS population are still unknown. To date, only one 22q11.2DS patient who died from complications related to AR aneurysm has been described in the literature [25].

Finally, our study highlighted that the risk of ARD is inversely related to BMI. Some scientific evidence has shown that patients with ARD have lower adipose and fat-free body masses than individuals with normal AR size [26]. Furthermore, in obese patients, a local vasoconstriction effect around the AR, mediated by perivascular adipose tissue, has also been hypothesized [27].

Limitations of our analysis include the relatively small sample size of patients enrolled in a single center, the limited length of the follow-up and the availability of the echocardiographic longitudinal data for only 14 out of 24 patients with ARD. In addition, a genetic characterization of the entire cohort could not be investigated due to the unavailability of genetic data of some patients.

## 5. Conclusions

Our study confirms that, in addition to the typical major intracardiac CHDs observed in 22q11.2DS patients, it is also important to investigate the possible presence of isolated ARD or in association with minor cardiovascular anomalies. Although most patients present a mild dilation, our longitudinal analysis showed a statistically significant increase in the absolute diameter of the AR in individuals with ARD over time. Assessment of ARD in terms of Z-score may be useful for the overall risk stratification, but being a parameter closely dependent on BSA, an increase in weight may lead to an error in assessment compared with an earlier control. In addition, adults with 22q11.2DS often experience significant changes in their body weight due to a tendency to overweight/obesity, DMT2 and possible side effects of neuropsychiatric therapies.

Although our study identified a significant direct relationship between ARD and the presence of skeletal and connective tissue disorders, its clinical implication and genetic explanation remain to be elucidated.

To date, a specific screening protocol for clinical practice regarding ARD in patients with 22q11.2DS has yet to be defined. Given the high prevalence of isolated ARD in this population and the limited data on the longitudinal course, a periodic cardiac evaluation for all patients with this genetic syndrome seems to be warranted.

Further studies on larger cohorts are needed to better clarify the clinical risk factors associated with ARD, to investigate the pathogenetic mechanisms underlying this condition and to evaluate the possible risk of complications in single patients.

## Figures and Tables

**Table 1:** Patients’ characteristics at enrollment.

	22q11.2DS without Major CHD (*n* = 74)	Non-ARD (*n* = 50)	ARD (*n* = 24)	*p* Value
**Male, *n* (%)**	44 (59.5)	27 (54)	17 (70.8)	0.167 ^1^
**Age (years), median (IQR)**	27.5 (23–35)	26.5 (23–32)	28.5 (22.5–39)	0.246 ^2^
**BMI (Kg/m²), median (IQR)**	23.7 (20.5–29.1)	22.2 (21.9–32.3)	21.45 (20.1–25.3)	**0.005** ^2^
**CV risk factors, *n* (%)**				
Dyslipidemia	7 (15.6)	5 (20)	3 (12.5)	0.867 ^1^
Hypertension	11 (14.9)	8 (19)	3 (12.5)	0.715 ^1^
Smoking	10 (17.8)	7 (19.4)	3 (15)	0.519 ^1^
DMT2	3 (4.0)	2 (4)	1 (4.1)	0.601 ^1^
Overweight/obesity	33 (44.6)	25 (50)	8 (33.3)	0.254 ^1^
**Aortic arch/epiaortic vessel anomalies, *n* (%)**	20 (27)	9 (18)	11 (45.8)	**0.012** ^1^
Double aortic arch	1 (1.4)	0	1 (4.2)	0.146 ^1^
Right aortic arch (isolated)	15 (20.3)	8 (16)	7 (29.3)	0.223 ^1^
Right aortic arch with aberrant left subclavian artery	8 (10.8)	4 (8)	4 (16.7)	0.424 ^1^
Left aortic arch with aberrant right subclavian artery	5 (6.8)	1 (2)	4 (16.7)	**0.019** ^1^
Kommerell diverticulum	3 (4.1)	1 (2)	2 (8.3)	0.196 ^1^
Vascular ring	2 (2.7)	1 (2)	1 (4.2)	0.591 ^1^
**Crossed pulmonary arteries, *n*(%)**	19 (25.7)	10 (20)	9 (37.5)	0.107 ^1^
**Bicuspid aortic valve, *n* (%)**	4 (5.4)	2 (4)	2 (8.3)	0.440 ^1^
**AAD, *n* (%)**	19 (25.7)	6 (12.2)	13 (54.2)	**<0.0001** ^1^
**Skeletal/Connective tissue disorders,** ***n* (%)**	56 (78.9)	33 (70.2)	23 (95.8)	**0.012** ^1^
Scoliosis	48 (67.6)	30 (63.8)	18 (75.0)	0.341 ^1^
Skeletal anomalies of lower limbs	28 (40)	17 (37)	11 (45.8)	0.472 ^1^
Skeletal anomalies of upper limbs	7 (10)	7 (15.2)	0	**0.044** ^1^
Vertebral abnormalities	7(10)	3 (6.5)	4 (16.7)	0.179 ^1^
Ligamentous laxity	3 (4.3)	1 (2.2)	2 (8.3)	0.227 ^1^

^1^ Pearson’s chi-squared test, ^2^ Wilcoxon rank sum test. CHD: congenital heart disease, ARD: aortic root dilation, AAD: ascending aorta dilation, CV: cardiovascular, DMT2: diabetes mellitus type 2.

**Table 2:** Prevalence, Z-score and severity of ARD and AAD in 22q11.2DS patients.

Variable	ARD	AAD
**Prevalence, *n* (%)**	24/74 (32.4)	19/74 (25.7)
**Degree, *n* (%)**		
Mild	16/24 (76.7)	13/19 (68.4)
Moderate/severe	8/24 (33.3)	6/19 (31.6)
**Z-score, median (IQR)**	2.43 (2.08–3.01)	2.7 (2.4–3.2)

ARD: aortic root dilation, AAD: ascending aorta dilation.

**Table 3:** Genetic data of the total cohort of 22q11.2DS patients.

		22q11.2DS without Major CHD (*n* = 74)		Non-ARD (*n* = 50)		ARD (*n* = 24)		*p* Value ^1^
		*n*	%	*n*	%	*n*	%	
Diagnostic test	FISH	26	35.1%	19	38.0%	8	33.3%	1.000
	SNP-/CGH-array	47	63.5%	31	62.0%	15	62.5%	
	MLPA	1	1.4%	0	0.0%	1	4.2%	
Segregation	De novo	18	37.5%	12	37.5%	6	37.5%	1.000
	Parental	2	4.2%	2	6.3%	0	0.0%	
	Unknown	28	58.3%	18	56.3%	10	62.5%	
22q11.2 deleted position	Proximal	43	89.6%	28	87.5%	15	93.8%	0.593
	Central	2	4.2%	2	6.3%	0	0.0%	
	Distal	3	6.3%	2	6.3%	1	6.3%	
22q11.2 deleted size	Typical (2.5–3 Mb)	35	72.9%	22	68.8%	13	81.3%	0.358
	Atypical (<2.5 Mb)	13	27.1%	10	31.3%	3	18.8%	
Additional CNVs	No	29	61.7%	20	62.5%	9	60.0%	0.777
	Yes	11	23.4%	8	25.0%	3	20.0%	
	Unknown	7	14.9%	4	12.5%	3	20.0%	

^1^ Pearson’s chi-squared test. CHD: congenital heart disease, ARD: aortic root dilation, CNVs: copy number variants.

**Table 4:** Risk factors associated with ARD in 22q11.2DS patients.

	B	*p* Value	OR	95% CI *per* OR
BMI (Kg/m²)	−0.14	**0.011**	0.86	0.77–0.97
Aortic arch/epiaortic vessel anomalies	1.11	0.076	3.02	0.89–10.27
Skeletal/Connective tissue disorders	2.55	**0.020**	12.82	1.43–115.31

## Data Availability

The data presented in this study are available on request from the corresponding author.

## Supplementary Materials

The following supporting information can be downloaded at: https://www.mdpi.com/article/10.3390/genes13122334/s1, Table S1: Clinical, genetic and echocardiographic data of 22q11.2 patients with ARD. Reference [28] is cited in the Supplementary Materials.
