# Supplementary material for: Clinical Risk Factors for Aortic Root Dilation in Patients with 22q11.2 Deletion Syndrome: A Longitudinal Single-Center Study
Source: Genes (Basel). 2022 Dec 10;13(12):2334. doi: 10.3390/genes13122334 (PMC9778342; doi:10.3390/genes13122334)
Supplement: Supplementary file 1 [file genes-13-02334-s001.zip › genes-1991572-supplementary.pdf]

Supplementary Table S1. Clinical, genetic and echocardiographic data of 22q11.2 patients with ARD

ID	Sex/ age	22q11.2 deletion type	Additional ~~mutations~~ CNVs (hg19 release)	CV risk factors	Aortic arch/ epiaortic vessels anomalies	Crossed Pulmonary Arteries	BAV	AR diameter (mm)	AR Z-score	ARD severity	AA diameter (mm)	AA Z-score	AAD severity	Skeletal/Connective tissue disorders
1	F/35	A-D, proximal	5q15(94786021_94856474)x1 pat (70 Kb); OMIM genes: *TTC37, FAM81B* – **VOUS***	Overweight	RAA	No	No	36.7	2.75	mild	22.4	0.18	No	Scoliosis, skeletal anomalies of lower limbs
2	M/50	A-D, proximal		No	No	No	No	33	3.24	moderate	30.5	4.03	Severe	Scoliosis, skeletal anomalies of lower limbs
3	M/ 23	A-D, proximal		No	Right aberrant subclavian artery in LAA	No	No	31.8	2.14	mild	27.6	2.59	Mild	Scoliosis, skeletal anomalies of lower limbs
4	M/ 24	NA		No	No	No	No	31.8	2.43	mild	22.5	1.6	No	Skeletal anomalies of lower limbs
5	F/46	A-D, proximal		Overweight	No	No	No	41.4	2.38	mild	24.94	1.41	No	Scoliosis
6	F/22	NA		No	RAA, left aber-rant subclavian artery	No	No	38	4.13	severe	26	2,66	Mild	Scoliosis
7	F/27	A-D, proximal		No	Right aberrant subclavian artery in LAA	Yes	No	33.1	2.11	mild	27.5	2	Mild	Skeletal anomalies of lower limbs
8	F/28	A-D, proximal		Smoking	No	Yes	No	35	3.18	moderate	21	1.01	No	Scoliosis
9	M/29	A-D, proximal		No	No	No	Yes	37	3.67	moderate	29.5	3.22	Moderate	Scoliosis
10	F/33	NA		Hypertension, smoking,	RAA	Yes	No	44.2	4.03	severe	36	2.66	Mild	Scoliosis, skeletal anomalies of lower

				overweight										limbs
11	F/21	NA		No	No	No	No	32	2.45	mild	20	0.79	No	Scoliosis, ligamentous laxity
12	F/51	A-C, proximal		Hypertension, dyslipidemia, DMT2, overweight	No	No	No	39	3.07	moderate	41	5.71	Severe	Scoliosis, skeletal anomalies of lower limbs
13	F/16	NA		No	No	Yes	No	31.8	2.84	mild	23.5	2.27	Mild	Skeletal anomalies of lower limbs, ligamentous laxity
14	M/54	A-C, proximal		Hypertension, dyslipidemia, overweight	No	No	No	34	2.55	mild	28	2.36	Mild	Scoliosis, skeletal anomalies of lower limbs
15	M/42	NA		Overweight	RAA, left aberrant subclavian artery, Kommerell diverticulum	Yes	No	35	2.26	mild	NA	NA	No	Scoliosis
16	F/27	A-D, proximal		Smoking	No	No	No	39	2.72	mild	32	1.63	No	Scoliosis
17	F/21	NA		No	Right aberrant subclavian artery in LAA	No	No	31.5	2.17	mild	24,6	1.97	No	Scoliosis
18	F/20	A-C, proximal		No	No	No	No	41.6	3.92	moderate	32.5	2.67	Mild	No
19	M/32	NA		No	RAA	Yes	Yes	32	2.39	mild	28.5	3.05	Moderate	Vertebral anomalies
20	F/43	A-C, proximal	2p25.3(813379_1973174)x3 mat (1.16 Mb); OMIM genes: *SNTG2, TPO, PXDN, MYT1L* - **VOUS***	No	DAA, RAA, left aberrant subcla-	No	No	33	3.01	moderate	19.46	0.91	No	Scoliosis, vertebral anomalies

					vian artery									
21	F/21	A-C, proximal		No	No	Yes	No	33	2.43	mild	25,2	1.92	No	Scoliosis, skeletal anomalies of lower limbs
22	F/32	A-D, proximal		Dyslipidemia, overweight	Right aberrant subclavian artery in LAA	Yes	No	38.4	2.51	mild	27	0.39	No	Vertebral anomalies
23	F/36	A-D, proximal	-7p22.3(1017098_1245755)x1 (229 Kb); OMIM genes: *CYP2W1, MIR339, GPER* - **VOUS***<br>-7q21.3(95358811_95473380)x3 (115 Kb); OMIM genes: *DYNC1I1* - **VOUS***	Overweight	RAA, left aberrant subclavian artery, Kommerell diverticulum	Yes	No	36	2.95	mild	30	2.67	Mild	Scoliosis, vertebral anomalies
24	F/24	D-E, distal		No	No	No	No	30.5	2.08	mild	27	2.85	Mild	Scoliosis, skeletal anomalies of lower limbs

CV: cardiovascular, BAV: bicuspid aortic valve, AR: aortic root, ARD: aortic root dilation, AA: ascending aorta, AAD: ascending aorta dilation, pat: paternally inherited, mat: maternally inherited, RAA: right aortic arch, LAA: left aortic arch, DAA: double aortic arch, NA: not available, VOUS: CNVs of uncertain significance. *All CNVs were classified according to ACMG (American College of Medical Genetics and Genomics) recommendations [1].

References Of Supplementary Table

1. Riggs, E.R.; Andersen, E.F.; Cherry, A.M.; Kantarci, S.; Kearney, H.; Patel, A.; Raca, G.; Ritter, D.I.; South, S.T.; Thorland, E.C.; et al. Technical standards for the interpretation and reporting of constitutional copy-number variants: a joint consensus recommendation of the American College of Medical Genetics and Genomics (ACMG) and the Clinical Genome Resource (ClinGen). *Genet Med* **2020**, *22*, 245-257.
